# Oral Microbiome Alterations After Cancer Treatment: A Scoping Review and Analysis

**DOI:** 10.21203/rs.3.rs-6930838/v1

**Published:** 2025-06-24

**Authors:** Francis A. Boksa, Leah I. Leinbach, Drashty P. Mody, Sukirth M. Ganesan, Jacqueline W. Mays

**Affiliations:** National Institute of Dental and Craniofacial Research, National Institutes of Health; National Institute of Dental and Craniofacial Research, National Institutes of Health; National Institute of Dental and Craniofacial Research, National Institutes of Health; National Institute of Dental and Craniofacial Research, National Institutes of Health; National Institute of Dental and Craniofacial Research, National Institutes of Health

**Keywords:** oral microbiome, cancer therapy, cancer survivorship

## Abstract

**Background:**

Cancer therapies impact the oral cavity. Oral microbial changes occur following cancer therapy, but the nature, duration and implications of these shifts are not well understood. Exposure to radiation, chemotherapy or cellular therapies has been associated with oral microbiome shifts toward dysbiosis and increased frequency of pathogenic species in the microbiome. Despite these findings, much remains unknown about cancer-therapy related changes in the oral microbiome following specific therapies, and what the associated long-term oral health implications may be for cancer survivorship. We therefore conducted a scoping review of oral microbiome studies in patients undergoing cancer therapy to broadly synthesize the literature on the oral microbiome in the context of cancer therapy, categorize findings, and identify research gaps to inform future projects.

**Results:**

This scoping review of the literature describes substantial changes in the oral microbiome during cancer treatment, specifically chemotherapy, radiation therapy, and stem cell transplantation. Across 62 studies, chemotherapy, radiation therapy, and stem cell transplantation were associated with significant changes in oral microbial communities. Among studies that assessed alpha diversity, the richness of species that comprised the oral microbiome often increased, encompassing more microbial taxa, while the evenness of species decreased, suggesting that community change was dominated by a few species. Frequently observed changes included decreases in commensal taxa such as *Streptococcus* and *Prevotella* and increases in opportunistic organisms such as *Candida albicans* and *Staphylococcus aureus*. While mucositis was often studied, consistent microbial predictors of this outcome were not identified

**Conclusions:**

The present study lays the groundwork for future research on the unique oral microenvironment and health needs in cancer survivors. The alterations in microbiome composition associated with specific cancer treatment categories add to our limited understanding of the key role of cancer therapy modalities on the oral microbiome.

## BACKGROUND

The oral microbiome includes bacterial, fungal, and viral species that colonize distinct niches of the oral cavity such as the periodontium, the tongue, and the teeth (1, 2). Typically, the oral microbiome remains naturally segregated from the gut microbial community, with microbial exchange among microbiomes impacting homeostasis and health (3–5). Microbial dysbiosis is broadly defined as the loss of beneficial microorganisms, overgrowth of potential or known pathogens, and a reduction in overall diversity (6). Cancer therapies can induce dysbiosis through direct cytotoxic effects or indirectly through altered immune function, accompanying antimicrobial prophylaxis, and reduced salivary flow. Sequelae of these therapies can include reduced saliva production, dysgeusia, oral mucositis, increase risk of infection related to immune suppression, and impact diet and nutritional deficiencies (7–12). These sequelae of treatment may contribute to disruptions in the oral microbiome, which in turn may impact not only oral health outcomes but also systemic complications via bloodstream infections, impaired treatment response, or direct communication with the immune system (13–16). The effects of such treatments on the oral microbiome remain relatively underexplored. Understanding these changes is critical, as oral microbiome disruptions may predispose patients to local and systemic infections, mucosal barrier injury, or compromised return to health.

Recent evidence suggests that the gut microbiome plays a role in anti-tumor immunity (ex: *F. nucleatum* and suppression of antitumor immunity) and response to common cancer treatments (17–19). For example, chemotherapy can induce microbial dysbiosis, altering immune and metabolic functions in the gastrointestinal tract (17). Emerging evidence suggests the gut microbiome may modulate the efficacy of immune checkpoint inhibitors (ICIs), with microbiome-targeted interventions showing potential to enhance ICI therapeutic response (20, 21). Whether similar effects occur in the oral cavity remains unknown, although given that the oral microbiome shares microbial characteristics with the rest of the gastrointestinal tract, it is reasonable to consider that oral microbiota may play similar role via alterations in mucosal immunity, systemic inflammation, and host-microbe interactions (22–26).

This scoping review synthesizes current evidence to examine the relationship between the oral microbiome and cancer therapies to identify areas for future research. There were three broad objectives: 1) to describe the types of cancer therapies assessed in relation to the oral microbiome; 2) to review techniques used and reported microbiome characteristics; 3) to assess the effects these therapies may have on the oral microbiome in cancer patients. We selected a scoping review format due to the anticipated heterogeneity in study design, patient population, sampling methods, and analytical approaches. Findings from this scoping review may guide future mechanistic studies and inform the development of targeted interventions to mitigate oral and systemic complications in patients undergoing cancer therapy.

## METHODS

The review protocol is registered at Open Science Framework (https://doi.org/10.17605/OSF.IO/4HNWS). The Preferred Reporting Items for Systematic reviews and Meta-Analyses extension for Scoping Reviews (PRISMA-ScR) checklist was used for reporting this completed review (25). This study was exempted from institutional review board approval due to the public nature of the data.

### Eligibility Criteria

All journal articles, abstract, randomized controlled trials (RCT), case reports, cohort studies, reviews, and expert commentary were considered for inclusion. Articles were included if they were: (1) concerning a population undergoing medical treatment for malignancy, (2) had at least one measure assessing changes in the oral microbiome due to cancer therapy or a change in the oral microbiome due to complications of cancer therapy, (3) published after January 1, 1990, and (4) published in English. Articles were excluded if they were: (1) published prior to 1990, (2) not published in English, (3) did not include an oral microbiome data/discussion, (4) only evaluated clinical oral complications without assessing microbial changes (i.e., mucositis, xerostomia), (5) only assessed microbial burden without discussing taxonomic identification, (6) reported patients undergoing therapy for any complication/ancillary issue related to cancer/cancer therapy as opposed to the cancer therapy itself, and (7) did not include human subjects data.

### Information Sources & Search Strategy

The database research was developed by a medical librarian (AAL) and further adapted and executed by a review team member (FAB). Databases searched included PubMed/MEDLINE (National Library of Medicine) and the Cochrane Library: Database of Systematic Reviews & CENTRAL (Central Register of Controlled Trials) (Wiley & Sons). The searches were limited to those published January 1, 1990, in the English language and animal studies and specific article types were excluded using a search strategy as per eligibility criteria. The searches were completed in August 2021 and updated searches in August 2023, and February 2025. See Supplemental File # for final search strategies used. Review and analysis occurred between August 2023 and March 2025.

### Screening

Covidence (Veritas Health Innovations, Melbourne, Australia) was used for screening. A two-stage screening process was completed. First, every article’s title and abstract were screened by two separate reviewers (FB, DM, JM) independently using the eligibility criteria. A consensus vote was required for each record, and conflicts were resolved by discussion between the reviewers until a consensus was achieved. Second, each record included after stage one proceeded to full text review where two reviewers (FB, DM, JM, LL) independently screened using the eligibility criteria. Conflicts were resolved by discussion between the reviewers until a consensus was achieved.

### Data Collection & Data Items

To extract the pertinent information from included articles, an online data extraction form was created and used in Microsoft Forms. Before extraction, four reviewers (FB, DM, JM, and LL) calibrated the process by completing the data extraction form together for the first included record and comparing results. Data were collected from each included article by a single reviewer (FB, DM, and LL). Spot checks were conducted on a subset of data extractions for quality control. Reconciliation of discrepancies was resolved through discussion and re-examination of the study and any supplemental material. The following data items were collected: study design and patient demographics, disease and treatment characteristics, microbiome sampling and classification methodology, statistical tests and analyses used, alterations to the oral microbiome, and relevant conclusions relating to the aims of this review. One reviewer (FB) extracted and compared specific microbiome data available from the individual studies across all included articles in which it was available.

### Synthesis of Results: Article Characteristics

The articles included were categorized according to key features including year published, study design, number of participants, participant age, country of origin, and funding source. Clinical characteristics included cancer type studied, cancer therapy, and oral sequalae studied (if any). Technical characteristics including sampling source (i.e. anatomic site from which the oral microbiome sample was obtained), types of microbes assessed, outcomes (i.e alpha diversity, beta diversity, change in specific species), and identification techniques were also categorized.

### Synthesis of Results: Taxonomic Analysis

To broadly synthesize results of each of the included studies, taxa identified as altered were extracted and pooled based on the type of cancer therapy, sample source, and taxonomic identification method. Change to the specific microbiome composition was assessed by calculating the sum of the number of times each reported taxon was gained or lost, defined as taxa that were either present or absent following cancer treatment but not in respective controls. Overgrowth of potential or known pathogens was assessed by calculating the sum of the number of times each reported taxon was increased or decreased, defined as taxon present in both treatment and control groups but at different abundance. Results at the level of phylum, order, and genus are reported. Because the depth of taxonomic assignment differed between included studies, all extracted microbes were assigned higher-rank classification up to the level of phylum using the National Center for Biotechnology Information (NCBI) taxonomy database so that accurate comparisons could be made (27, 28).

### Synthesis of Results: Statistical Analysis

Frequencies and percentages were calculated for all categorical variables, means and standard deviations for continuous variables using Microsoft Excel. GraphPad Prism (Boston, MA) was used for comparative taxonomic analyses.

## Results

### Study Characteristics

**Figure 1:**
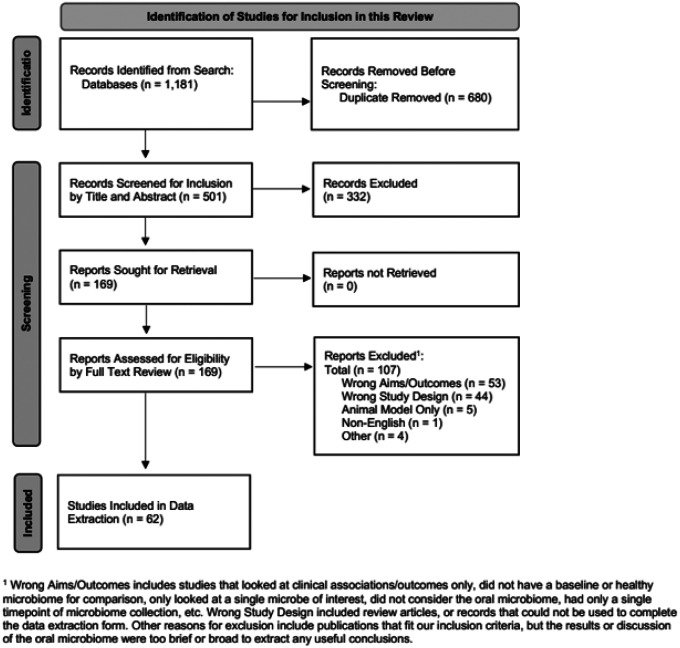
PRISMA Flow Diagram PRISMA flow diagram for scoping reviews detailing the results of the systematic search strategy, record screening, and the number of publications considered for final inclusion in this review.

Five hundred and one unique articles were identified for title and abstract screening. Of these, 169 articles were considered for full text review, and 62 ultimately met inclusion criteria for data extraction and analysis (Figure 1). Twenty-five articles (40%) were published in 2020 or after, 11 (18%) before 2010 (Table 1). Twelve (19%) were by authors from the United States, 8 (13%) from Brazil, and 7 (11%) from Japan. Most were observational studies (94%) with an average study size of 57 participants (SD: 42), a majority of which were exclusively adult populations (76%, n=47). Forty-five percent (n=28) received at least partial government funding.

### Sampling Techniques & Classification

The oral mucosa was the most common niche sampled (n=34), with bacteria the most common taxa examined (65%, n=40) (Table 3). Fewer studies investigated multiple organisms (i.e., bacteria, fungi, and viruses) (26%, n=16); 51.6% of studies investigated alpha diversity (n=32), while beta diversity was less common (25.8%, n=16). All studies assessed some level of change in total oral microbiota; 58% (n=36) examined common oral sequalae of cancer therapy (i.e. oral mucositis, candidiasis, xerostomia). Differential culture and phenotyping were the most common techniques used (46.8%, n=29), followed by rRNA gene sequencing (45.2%, n=28), with metagenomic sequencing as the least common (3.2%, n=2).

### Disease & Treatment Characteristics

Most studies (82%, n=51) investigated the oral microbiome in patients with specific types of cancer (Table 2). Of this, 57% were hematologic malignancies (n=29) and 34% were head and neck cancers (n=17), with colorectal, breast, and lung the other cancer types represented. Chemotherapy was the most common treatment modality investigated among all studies (40.3% of studies, n=25), followed by hematopoietic cell transplantation (HCT, 25.8%, n=16) and radiation therapy (25.8%, n=16). Only one study assessed the oral microbiome in patients receiving immunotherapy, in this case for lung cancer.

Among the 25 studies examining chemotherapy, 60% (n=15) involved adults and 36% (n=9) included pediatric patients (Table 2). Over half (52%, n=13) focused on hematologic malignancies, while others involved mixed or unspecified cancer types. Eight studies evaluated mucositis; five of which examined alpha and beta diversity. Of the sixteen studies that assessed the oral microbiome in relation to radiation therapy, most (81% n=13) focused on adult patients with head and neck cancer. Eight studies focused on oral complications common after radiation therapy – namely mucositis (n=3), hyposalivation (n=2), dental caries (n=2), and periodontal disease (n=1). All 16 HCT studies examined patients with hematologic malignancies. Most (82%, n=13) were adult cohorts; 69% (n=11) focused on mucositis.

### Pooled Taxonomic Analysis

#### Changes in Overall Frequency

**Figure 2:**
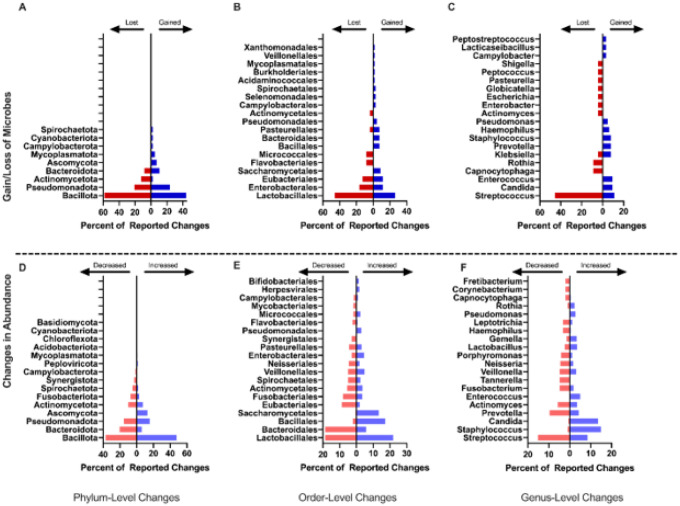
Combined Most Frequent Microbiome Changes Pooled results from the 62 records included in this review detailing the most frequently altered microbial taxa with cancer therapy. Results are separated by taxonomic rank and the levels of phylum (A, D), order (B, E), and genus (C, F) are reported, and by the type of change, with gained/lost (A-C) microbes indicating that a taxon was present or absent following cancer treatment but not in controls, and increased/decreased (D-F) indicating that the taxon was present in both groups, but at significantly different abundance. Percentages were calculated for each taxonomic level and type of change. At the level of phylum, there was a combined total of 84 instances of gained taxa, 24 lost, 471 increased, and 403 decreased. At the level of order there were 69 taxa gained, 24 lost, 449 increased, and 376 decreased, and at the genus level there were 64 taxa gained, 22 lost, 422 increased, and 336 decreased.

All sixty-two studies included some level of taxonomic analysis. A pooled taxonomic summary is reported in Figure 2. At the phylum level, Bacillota and Pseudomonadota were the most commonly reported in relation to changes in overall diversity with gains in 44% and 24% and losses in 58% and 21% of events, respectively (Figure 2A). At the order level, changes in Lactobacillales, Enterobacterales, and Eubacteriales were most commonly reported in relation to changes in overall diversity with gains in 26.1%, 11.6%, and 11.6% of events, and losses in 45.8%, 16.7%, and 12.5% of events respectively (Figure 2B). At the genus level, losses of *Streptococci* were most common (45.5% of events), although gains were reported in 10.9% of events (Figure 2C). Known pathogens or potentially pathogenic genera (i.e. *Candida*) were most commonly reported as gained (9.4% of events; Figure 2B). Organisms commonly associated with health such as *Rothia* and *Actinomyces*) were reported as lost in 9% of available events. Genera associated with both health and disease including *Streptococcus and Klebsiella* were most reported as both gained in 10.9%, and 7.8% of events, and lost in 45.5% and 4.5% of events, respectively.

#### Changes in Abundance

In addition to the gain and loss of specific taxa, studies also reported changes in abundance of taxa. Bacillota, Pseudomonadota, and Bacteroidota were the phyla most reported as changed in relative abundance with both increases in 47.8%, 15.7%, and 5.9% of events, respectively, and decreases in abundance in 36.7%, 20.3%, and 15.4% of events, respectively, reported in available studies (Figure 2D). Members of the order Lactobacillales, Bacteroidales, and Bacillales were most commonly reported as changed (Figure 2E). Most studies reported a decrease in Bacteroidales (18.6% of reported events) and an increase in Bacillales (17.1% of events) with reports of Lactobacillales members changing in both directions, with increased frequency in 22.0% of events and decreased frequency in 18.6% of events, indicating an order impacted by cancer therapy through changes in multiple species. Known pathogens or potentially pathogenic genera were most commonly reported as increased including *Candida* (13.5% of events)and *Staphylococcus* (14.9% of events; Figure 2F). Genera commonly associated with a healthy oral microbiome were more frequently reported as reduced, including *Prevotella* (9.5% of events)and *Veillonella* (4.8% of events; Figure 2F).

#### Changes By Treatment Type

##### Chemotherapy

Of the 62 studies included in this analysis, 25 (40.3%) were studies investigating the relationship between the oral microbiome and chemotherapy. In these studies, mucositis was associated with more pronounced microbial shifts, including reductions in diversity (Ye, Klymiuk) and shifts in composition pre- and post-therapy (Omori)(29). Two studies (Hong, Singh) found enrichment of *Fusobacterium nucleatum* during chemotherapy regimens that included 5-fluorouracil in some but not all patients (30, 31). Other studies varied in focus. Aitken targeted *Stenotropomonas maltophilia*, while Arastehfar, Diaz, and Sun studied candidiasis (32–34). Broader surveys (Napenas, Galloway-Pena, Beier Jensen, Franklin, Proc) revealed no consistent themes but highlighted the emergence of previously undetected species (Napenas), associations between community variability and poor outcomes (Galloway-Pena), and decreases in *Prevotella* balanced by increases in *Campylobacter, Fusobacterium*, or *Neisseria* species following broad-spectrum antibiotics (Franklin) (35–39).

**Figure 3:**
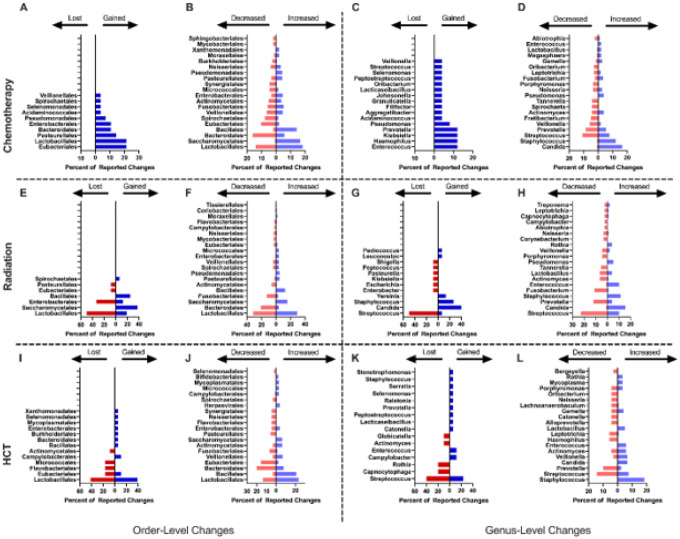
Most Frequent Microbiome Changes by Treatment Type Results from the 62 included records were stratified and pooled based on the treatment modality. Results for each treatment type, chemotherapy, radiation therapy, and hematopoietic cell transplantation, are separated by taxonomic rank, and the levels of Order (A, B, E, F, I, J) and Genus (C, D, G, H, K, L) are reported, and by the type of change, with gained/lost (A, C, E, G, I, K) and increased/decreased (B, D, F, H, J, L) taxa reported. Chemotherapy (A-D) results are pooled from 25 records and at the level of order there are combined instances of 28 taxa gained, 0 lost, 191 increased, and 156 decreased. At the level of genus there are 25 taxa gained, 0 lost, 181 increased, and 139 decreased. 16 records investigated radiation therapy (E-H), with a combined total of 16 taxa gained, 12 lost, 103 increased, and 95 decreased at the order level and 15 gained, 12 lost, 97 increased, and 92 decreased taxa at the level of genus. Additionally, 16 records investigated hematopoietic cell transplantation (I-L) with a pooled total of 18 gained, 12 lost, 129 increased, and 89 decreased taxa at the level of order, and 17 gained, 10 lost, 119 increased, and 70 decreased taxa at the level of genus.

Pooled taxonomic analysis of the records investigating chemotherapy did not reveal any lost taxa, however, there were several gained taxa (Figure 3A). At the level of Order, the most frequently gained taxa were members of Eubacteriales (21.4% of events), Lactobacillales (21.4% of events), and Pasteurellales (14.3% of events). In addition, many taxa showed an altered abundance following chemotherapy (Figure 3B). Members of Lactobacillales accounted for 18.3% of increased taxa and 14.1% of decreased taxa. Other commonly increased Orders include Saccharomycetales (16.7% of events) and Bacillales (14.1% of events), while the most frequently decreased taxa belong to the Orders Bacteroidales (16.0% of events) and Eubacteriales (10.3% of events). The genus level showed gains in *Haemophilus, Klebsiella, Prevotella*, and *Enterococcus* (each accounting for 12% of reported events; Figure 3C). An increase in abundance of potentially pathogenic genera was observed for *Staphylococcus* (7.7% of reported events)and *Candida* (16.6% of reported events). Organisms associated with microbiome health, such as *Rothia*, showed mixed results with studies reporting both increases and decreases in abundance related to chemotherapy treatment. Other organisms associated with health such as *Gemella* often increased in abundance (2.8% of events; Figure 3D). Organisms associated with both health and disease (*Streptococcus, Actinomyces, Veillonella, Prevotella*) were reported as both increased in 7.7%, 3.9%, 1.7% and 5.5% of events, and decreased in 10.8%, 4.3%, 5.8%, and 8.6% of events, respectively.

##### Radiation

Sixteen studies (25.4%) assessed the relationship between the oral microbiome and radiation therapy (40–55). Overall, a shift toward dysbiosis and a more pathogenic oral microbiome, was observed with increases in species such as *C. albicans* (n=8), *S. mutans* (8), and *R. dentocariosa* (n=4), and decreases in protective species such as *S. oralis* (n=6) and *S. sanguinis* (n=6) commonly reported. Increases in pathogenic taxa such as *Candida albicans, Enterococcus faecalis, Prevotella intermedia, Rothia dentocariosa, Streptococcus mutans, Staphylococcus aureus*, and *Streptococcus pneumoniae were* reported. Conversely, some studies observed declines in these same species. Reductions in commensal organisms such as *Streptococcus salivarius* (n=4) and *Streptococcus oralis* (n=5) were also noted, as were occasional decreases in pathogens like *Treponema denticola* and *Porphyromonas gingivalis*.

In contrast to chemotherapy, pooled taxonomic analysis of records investigating therapeutic radiation showed losses in members of the order Lactobacillales (50.0% of events) and Enterobacterales (33.3% of events), among others (Fig. 3E). Gains in Saccharomycetales (37.5% of events), Bacillales (25% of events) and to a lesser extent Lactobacillales (18.8% of events) were also observed (Figure 3E). Unlike chemotherapy, there were only a few orders comprising the majority of changes in abundance. Members of the order Lactobacillales were reported as mixed (increased in 29.1% of events, decreased in 31.6% of events). Members of the order Bacteroidales were decreased (21.1% of events), while Saccharomycetales were increased (15.5% of events; Figure 3F). Similar to findings observed after chemotherapy, members of the genus *Candida* were commonly reported as both gained after treatment and increased in abundance (40.0% of events, increased in 15.5% of events respectively). *Streptococci* were also reported as both lost (50.0% of events) and decreased (22.8% of events), *Staphylococci* as gained (26.7% of events) and increased (11.3% of events), and *Prevotella* as decreased (12.0% of events), similar directionally to findings related to chemotherapy (Figure 3G–H).

##### Hematopoietic Cell Transplantation (HCT)

Sixteen studies (25.4%) assessed the oral microbiome and HCT. Of these, healthy controls exhibited greater diversity (Badia), while reduced diversity and enrichment of pathogenic organisms was observed after HCT (Heidrich, Laheij, Faraci)(56–58). Mucositis severity was associated with the magnitude of microbiome change (Takahashi), and several studies described therapy-induced compositional shifts (Shouval) (59). Similar to Franklin *et. al*. in patients with acute leukemia after broad spectrum antibiotics, Rashidi *et. al*. suggested a degree of coalescence among the oral and fecal microbiomes in cancer survivors after HCT (60). Pooled taxonomic analysis at the genus level showed gains in *Campylobacter* and *Enterococcus* (both accounting for 11.8% of gained genera), with losses of *Rothia* and *Capnocytophaga* (both accounting for 20% of reported lost genera), among others. An increase in abundance of potentially pathogenic organisms including *Staphylococcus* (18.5% of events)and *Candida* (6.7% of events) was also observed.

Pooled taxonomic analysis of the records investigating HCT showed slight gains in members of several orders including Bacillales, Bacteroidales, and Enterobacterales (5.5% of events each), among others. Mixed results were observed regarding members of the order Lactobacillales (38.9% of gained events and 41.7% of lost events) and Eubacteriales (11.1% of gained events and 16.7% of lost events; Figure 3I). At the level of order, changes in the prevalence of taxa were similar to the trends observed in gained and lost taxa. Bacillales was frequently increased (20.9% of events), while Lactobacillales accounted for 23.3% of increased events and 13.5% of decreased events. Other commonly increased orders included Veillonellales (7.0% of events), Actinomycetales (6.2% of events), and Saccharomycetales (6.2% of events). Unlike the trend observed in gained taxa, Bacteroidales was the most frequently decreased order (20.2% of events), suggesting existing species within this order may be replaced by less common inhabitants of the oral microbiome. Other frequently decreased orders following HCT include Eubacteriales (15.7% of events), Fusobacteriales (6.7% of events), and many other minor contributors (Figure 3J).

Similar in directionality to findings in both chemotherapy and radiation, members of the genus *Streptococcus* were most commonly reported as lost (40.0% of events) and decreased in abundance (14.3% of events). *Staphylococci* were reported as gained (5.9% of events) and increased (18.5% of events), and *Prevotella* as decreased (10.0% of events), again similar in directionality to findings in chemotherapy and radiation (Figure 3K–L). 10 studies assessed changes at the species level. Common themes included elevated presence of opportunistic pathogens such as *Candida spp*. (n=10), *P. gingivalis* (n=4), and *C. gingivalis* (n=2).

## DISCUSSION

There is consistent evidence for a relationship between cancer therapies and changes in the oral microbiome, although the mechanism or mechanisms through which this occurs is not well-defined. Several themes emerged from this scoping review of the oral microbiome and cancer therapies. One, chemotherapy, radiation, and hematopoietic stem cell transplantation were the main cancer therapies studied in relation to the oral microbiome. To date, immune checkpoint inhibitors and the oral microbiome were not well-studied and appear to be an opportunity for future research. For example, mucositis related to immune checkpoint inhibitors has been reported in 3–4% of patients, and can manifest as oral pain and subjective dryness, and may be related to microbial changes similar to those described in the gut (61). Findings from this scoping review show that oral mucositis has been frequently studied in relation to the oral microbiome. It can lead to weakening of the mucosal barrier, increasing the risk of oral-source bacteremia/septicemia which can be fatal particularly in immune compromised patients. Among the 22 studies in this scoping review that assessed mucositis, no major themes emerged.

Second, techniques used to assess the oral microbiome were variable and changed with time. Earlier studies focused on specific oral species while later studies examined alpha and beta diversity. Still, most studies focused on bacterial species; inclusion of other microorganisms such as viruses and non-candida fungi could be an opportunity for further research. Additionally, studies investigating the microbiome as a whole, including bacteria, fungi, and viruses together, are lacking in the current literature. Future work investigating the changes and relationship between these domains could provide a more complete description of how the microbiome changes with cancer therapy.

Third, a broad assessment of the effect of cancer therapies on the oral microbiome suggests that among studies that assessed alpha diversity (intra-sample), the richness (i.e. the number of species) comprising the oral microbiome often increases while the evenness (i.e. the distribution of species abundance) decreases. This suggests that while the total number of microbes within a specific sample may increase after cancer therapy, the increase is driven by changes in relatively few organisms. Assessment of beta diversity (between-sample) was less commonly assessed. Among studies that included beta diversity, cancer treatment cohorts became more heterogeneous, with an increase in intra-group variability. Given that many of the changes observed were decreases in commensal microbes that commonly inhabit the healthy oral sites and increases in opportunistic pathogens, this is not unexpected. These kinds of shifts would be heavily dependent on many factors like their presence and levels at the start of treatment, interactions with other microbes, immune response, specific niche in the oral cavity, and many other factors that may potentially favor some species in one person/site and other species in another person/site. Assessing the pathogenicity of commonly altered organisms yielded mixed results. One common theme across the studies was decreases in several commensal and health associated microbes, such as *S. sanguinis, S. salivarius and S. oralis*, among others. These species are common inhabitants of the healthy oral microbiome may play a protective role by moderating pH, preventing inflammation, and inhibiting pathogenic species from colonizing oral sites (15, 62, 63).

Another common theme that emerged was an increase in the prevalence of pathogenic species and opportunistic pathogens such as *Candida spp., P. gingivalis, E. faecalis*, and *R. dentocariosa*. These species have been well documented to cause pathology within the oral cavity, including oral thrush, and are frequently associated with conditions such as caries and periodontitis. Many of the gained microbes, such as *P. aeruginosa, H. influenzae, K. oxytoca, S. aureus*, and others are not commonly found in the oral cavity, but are common inhabitants of other sites such as the gastrointestinal tract (64–66). Other studies report identification of presumed oral microbiome species in the fecal microbiome, suggesting a breakdown of natural barriers between the oral and gastrointestinal microbiomes. This could be related to the actual cancer therapy agents or may be related to barrier tissue damage or immunosuppression subsequent to treatment. The clinical implications of this barrier breach are not clear and could be a target for future meaningful studies. Taken together, these findings may demonstrate a shift in the oral microbiome from a stable, health associated state to a dysbiotic one following cancer therapy. Oral dysbiosis may be indicated by a decrease in health-associated microbes and their replacement by known oral pathogens, in addition to oral colonization with microbes not typically associated with the oral cavity. Conversely, some taxa, such as *F. nucleatum* and *P. gingivalis* were frequently reported as increased or decreased in different studies, and no conclusions can be drawn relating to the impact of cancer therapy on these common taxa.

The health implications of coalescence between the oral and gastrointestinal microbiomes have been postulated but are not well-established. Predictive factors in the oral microbiome of the response to cancer therapies or of altered risk of serious complications of cancer therapy such as cGVHD following allogeneic HCT have been investigated but require further careful studies to reach a clinically actionable consensus. Future studies should aim to control for confounding variables such as dietary changes, oral hygiene practices, and antibiotic use, and should include carefully curated patient populations across cancer types and treatment regimens. A goal for this area of work would be to employ mechanistic studies linking microbiome alterations to clinically relevant outcomes in order to build interventional trials testing microbiome-modulating strategies with the ultimate goal of translating these insights into enhanced patient care. How changes in microbial composition and microbial metabolites interact with oncologic therapies and the immune system is underreported for the oral microbiome but has emerged as a clinically meaningful area for the gut microbiome (67).

This study has several important limitations. One, we present here an examination of the oral microbiome by broad cancer treatment type and, as such, it is not possible to attribute to any one therapy changes in the oral microbiome since patients are typically exposed to more than one therapeutic type. Two, the review was limited to studies published in English, meaning the non-English literature was excluded. Three, pooling data from differing taxonomic ranks necessitated populating all higher taxonomic ranks for comparison. Comparing these ranks can show some trends about microbiome changes, but the overall impact can be challenging to describe as different members of the same rank may be associated with health or disease. This methodology means that we couldn’t compare the number of studies that reported a change, except for at a species level, and reported the combined number of changes at each level. For example, in Fig. 2F where *streptococcus* is both one of the most commonly increased and decreased genera is not necessarily a conflicting result, as different species within *streptococcus* may be increased or decreased. Finally, only two studies in this dataset used metagenomic sequencing to assess the oral microbiome. As metagenomic sequencing can give a more complete perspective of the oral microbiome with less bias compared to other methods, the use of this and other meta-omic approaches could elicit more directly comparable and possibly more reproducible findings in future longitudinal studies of the oral microbiome.

## Conclusions

This scoping review highlights the significant but complex impact of cancer therapies and alterations in the oral microbiome, underscoring a general shift from a health-associated microbiome to a dysbiotic state characterized by a contraction of commensal microbes and an expansion in opportunistic pathogens. However, variability in study design, assessment techniques, and patient populations limits the ability to draw definitive conclusions about specific mechanisms or clinical oral health implications for cancer survivors. Further research to address gaps in the literature should employ longitudinal meta-omic approaches to capture both taxonomic and functional changes in the oral microbiome throughout cancer therapy.

## Supplementary Files

This is a list of supplementary files associated with this preprint. Click to download.

- OralMicrobiotaScopingReviewManuscript19Jun2025supplement.docx

## Figures and Tables

**Table 1:** Article Characteristics Summarizing characteristics of included articles in terms of year published, study design, study size, participant age, country of origin, and funding source.

Characteristic	Frequency (n=62)	Percent (%)
**Year Published**		
2020 or newer	25	40.3
2010–2019	23	37.1
Before 2010	11	17.7
**Study Design**		
Observational	58	93.5
Interventional	4	6.5
**Study Size (# of Participants)**		
Mean (SD)		
Median	57 (42)	-
Range	49	-
	3–209	-
**Participant Age**		
Adult	47	75.8
Pediatric	11	17.7
Mixed	4	6.5
**Country of Origin**		
USA	12	19.4
Brazil	8	12.9
Japan	7	11.3
China	6	9.7
India	5	8.1
Netherlands	5	8.1
Sweden	4	6.5
Poland	4	6.5
Other^1^	11	17.7
**Funding Source**		
Government^2^	28	45.2
Other	12	19.4
None	22	35.5

1 Includes articles from Germany (n=1), Denmark (n=2), Austria (n=1), Israel (n=1), Canada (n=1), France (n=1), Iran (n=1), Italy (n=2), and Thailand (n=1)

2 Includes articles with any government funding; Other funding sources include industry, academia, and foundation grants.

**Table 2:** Disease & Treatment Characteristics Summarizing disease characteristics for each of the studies, including cancer type, treatment modality, and specific oral sequalae investigated.

Characteristic	Frequency (from n = 62 papers)	Percent (%)
**Cancer Type**		
Head and Neck	17	27.4
Hematologic	29	46.8
Other^1^	5	8.1
Nonspecific	9	14.5
**Cancer Therapy**		
Chemotherapy	25	40.3
Radiation	16	25.8
HCT	16	25.8
Chemoradiation	4	6.5
Immunotherapy	1	1.6
**Oral Sequelae**		
Mucositis	22	35.5
Hyposalivation or Dysgeusia	5	8.1
Candidiasis	6	9.7
GVHD	3	4.8
Periodontitis or Caries	4	6.5
Multiple Sequalae	8	12.9
Nonspecific^2^	15	24.2

Abbreviations: GVHD=Graft-versus-Host Disease

1 Other includes retinoblastoma, breast, colorectal, and lung cancers; ^2^nonspecific includes multiple cancer types or articles in which cancer type was not defined

2 Nonspecific or Other includes studies in which no specific oral sequalae was studied

**Table 3:** Oral Microbiome Characteristics

Characteristic	Frequency (from n=62 papers)	Percent (%)
**Oral Microbiome Sample Source** ^1^		
Saliva	20	32.3
Oral Mucosa Swab	33	53.2
Dental Plaque	7	11.3
Oral Rinse	10	16.1
Other^2^	3	4.8
**Microbes Assessed**		
Bacteria	40	64.5
Fungi	5	8.1
Viruses	1	1.6
Bacteria, Fungi	12	19.4
Bacteria, Fungi, & Viruses	4	6.5
**Outcomes Assessed**		
Change in Total Oral Microbiota	62	100
Event Gains (Phylum/Order/Genus)	84/69/64	-
Event Losses (Phylum/Order/Genus)	24/24/22	-
Event Increases (Phylum/Order/Genus)	471/449/422	-
Event Decreases (Phylum/Order/Genus)	403/376/336	-
Alpha Diversity	32	51.6
Beta Diversity	16	25.8
Microbe Antibiotic Susceptibility	5	8.1
Other^3^	11	17.7
**Microbial Identification Techniques**		
rRNA Gene Sequencing	28	45.2
Metagenome Sequencing	4	6.5
Differential Culture & Phenotypic Methods	29	46.8
PCR&RFLP	8	12.9
MALDI-TOF Mass Spectrometry	4	6.5

Abbreviations: PCR=Polymerase Chain Reaction, RFLP=Restriction Fragment Length Polymorphism, MALDI-TOF=Matrix-Assisted Laser Desorption/Ionization Time-or-Flight

1 Some manuscripts included for review contained data from multiple oral sampling sources or used a combination of approaches to identify microbial taxa.

2 Other sample sources include periodontal pocket, tongue imprint, and fecal collection.

3 Other outcomes assessed include oral immune response, microbial interactions and metabolomics.

## Abbreviations

HCT: Hematopoietic cell transplantation
RCT: Randomized Controlled Trial
PRISMA-ScR: Preferred Reporting Items for Systematic reviews and Meta-Analyses extension for Scoping Reviews
ICIs: Immune Checkpoint Inhibitors
NCBI: National Center for Biotechnology Information
